# Response Rate, Acceptability and Effectiveness of an Intervention Offering HIV/STI Testing Via Apps Among Gay, Bisexual, and Other Men Who Have Sex With Men in Barcelona, Spain, from 2016 to 2020

**DOI:** 10.1007/s10461-023-04165-2

**Published:** 2023-09-28

**Authors:** Miguel Alarcón Gutiérrez, David Palma Díaz, Mireia Alberny Iglesias, Rafael Ruiz Riera, Rafael Guayta-Escolies, Patricia García de Olalla, Cristina Rius Gibert

**Affiliations:** 1 Department of Paediatrics, Obstetrics and Gynaecology and Preventive Medicine, Universitat Autònoma de Barcelona, Barcelona, Spain; 2 Epidemiology service, Agència de Salut Pública de Barcelona, Barcelona, Spain; 3 Centre of Epidemiological Studies of HIV/AIDS and STI of Catalonia (CEEISCAT), Health Department, Generalitat de Catalunya, Badalona, Spain; 4 Department of International Health, Care and Public Health Research Institute – CAPHRI, Faculty of Health, Medicine and Life Sciences, Maastricht University, Maastricht, The Netherlands; 5 Centro de Investigación Biomédica en Red de Epidemiología y Salud Pública (CIBERESP), Madrid, Spain; 6 Health Department, Catalan Institute of Health, Generalitat de Catalunya, Barcelona, Spain; 7 Direcció Estratègica d’Atenció Primària i Comunitària, Health Department, Generalitat de Catalunya, Barcelona, Spain; 8 Projects and Research Directorate, Council of Pharmacists Colleges of Catalonia, Barcelona, Spain; 9 Institut de Recerca de l’Hospital de la Santa Creu i Sant Pau, Institut d’Investigació Biomédica Sant Pau, Barcelona, Spain

**Keywords:** Men who have sex with men, HIV testing, STI testing, Health promotion, Dating apps

## Abstract

We evaluated the response rate, acceptability, and effectiveness of a preventive programme offering rapid HIV and other STI testing, as well as sexual counselling to gay, bisexual, and other men who have sex with men (GBMSM) via dating apps over a 4-year period. The programme was carried out in 9 out of the 10 districts in the city of Barcelona, Spain. The response rate was defined as the proportion of people responding to the message sent, acceptability as the proportion of those responding favourably, and effectiveness as the proportion of users requesting a test. We performed univariate analysis and multivariate logistic regression in relation with the response rate, acceptability and effectiveness. A total of 5,254 messages were send to different users. The response rate was 33.1% (n = 1,741), acceptability was 86.2% (n = 1,500), and effectiveness was 10.1% (n = 532). The factors associated with user response were recent connection to the app (aOR = 1.85; CI:1.39–2.46) and the presence of a profile photograph (aOR = 1.34; CI:1.11–1.64). Acceptability was associated with recent connection to the app (aOR = 1.98; CI:1.09–3.58). Effectiveness was associated with lower reported age (aOR = 0.98; CI:0.97–0.99), contact before 14:00 (aOR = 2.47; CI: 1.77–3.46), and recent connection to the app (aOR = 4.89; CI:1.98–12.08). Effectiveness was also greater in districts that were more disadvantaged or had fewer sexual health services. This study demonstrates that the use of these apps is an acceptable and effective method of prevention and sexual health promotion in GBMSM in this setting and identifies the associated factors that could guide such interventions.

## Introduction

In the last 40 years, gay, bisexual, and other men who have sex with men (GBMSM) have been found to have multifactorial vulnerability, increasing their morbidity and mortality due to HIV and the incidence of other sexually transmitted infections (STI) [1–4]. Consequently, The World Health Organization (WHO) and the Joint United Nations Program on HIV/AIDS (UNAIDS) have characterised this group as a collective that benefits from specific risk-reduction interventions [5].

GBMSM have been pioneers in the use of new technologies for sexual purposes [6]. Among them, geosocial networking applications (GSN apps, or dating apps) for smartphones are widely used in this population [7, 8]. GSN apps use the global positioning system (GPS) to facilitate personal encounters according to geographical proximity.

Several researchers have described the use of mobile technologies to facilitate access to prevention and sexual health promotion messages among key populations, such as GBMSM (mHealth) [9]. Among these, the use of GSN apps has been reported to be an effective way to reach these populations, with good acceptance and effectiveness in spreading preventive messages, offering screening services, and acting as a link to the health system [9–12].

The massive use of GSN apps has been reported to be a factor associated with the rapid spread of STIs, including HIV infection [11]. The prevalence of risky sexual behaviours has been reported to be higher among GBMSM using these apps than among GBMSM not using these apps. These behaviours include unprotected sex, drug use to maintain sexual relations (chemsex), and having multiple sexual partners [11, 13–16]. Explanations for this increased risk include the desired gratifications motivating these encounters (physical, social, psychosocial) and the proximity and immediacy of encounters arranged through these apps [17, 18]. Prior research has reported that attraction to online encounters can be particularly enhanced among GBMSM due to the safety and anonymity of this setting versus greater discrimination and stigma that may be present in offline or public settings [11].

In 2016, our research team conducted a 3-month pilot intervention to determine the response rate and acceptability of a programme offering HIV, syphilis, and hepatitis C testing through GSN apps for GBMSM in the city of Barcelona, Spain. The intervention was performed through commercially available apps, such as Grindr, Wapo and Planet Romeo, and showed a high response rate and acceptance [12]. However, the pilot intervention was conducted in a very small geographical area of the city, thus failing to capture area- and housing-related social inequalities [19]. Findings in the pilot programme led to new questions about the efficacy of this strategy in a more socially and geographically diverse group of GBMSM in the city. The aim of this study was to assess the response rate, acceptability, and effectiveness of a programme offering rapid HIV, syphilis, and hepatitis C testing via apps for GBMSM in different areas of the city of Barcelona, Spain, over a 4-year period.

## Methods

### Intervention and Variables

**Fig. 1:**
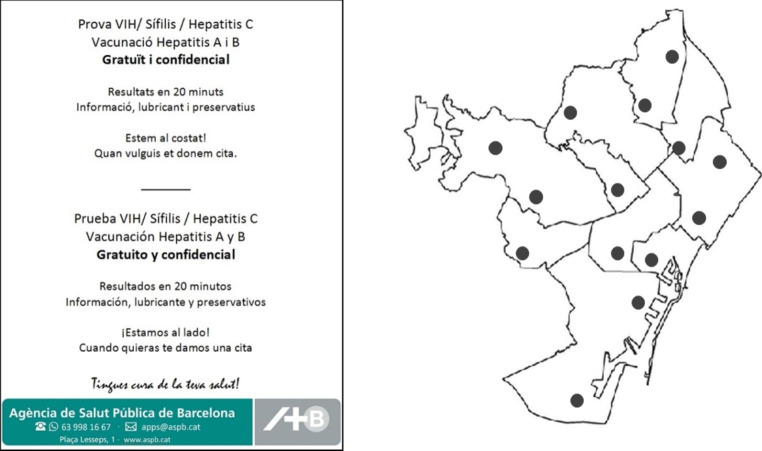
Sent message to app users and map of the city of Barcelona with intervention points among different districts

We conducted a descriptive study in the city of Barcelona between January 2016 and February 2020. GBMSM were contacted through the Grindr, Wapo, and PlanetRomeo apps. The research team sent messages through the profiles of users who were connected between 9:00 and 19:00 h, from Monday to Friday. The message, in the form of an image (Fig. 1), offered free and confidential rapid HIV, syphilis, and hepatitis testing. Users expressing a willingness to be tested could choose to attend the testing site immediately or could schedule an appointment for another time. The programme also offered online counselling on sexual health, covering mainly issues of sexual risk practices and information on the risk of drug use.

To increase the coverage area, the intervention was conducted in primary care centres in 9 of the 10 districts of the city (Fig. 1). For each contact made, we recorded self-reported age in years, the time of the intervention (before 14 h, between 14 and 17 h, and after 17 h), the time of the user’s last connection (as the apps show this information on users’ profiles in different timings: we considered in the last hour or more than 1 h ago), the presence of a user photograph (with or without a photo), the app used (Grindr, Planet Romeo, Wapo) and the district of the centre sending the message (Ciudat vella, Eixample, Sants-Montjuic, Sarrià-Sant Gervasi, Gràcia, Horta-Guinardó, Nou Barris, Sant Andreu, Sant Martí). The socioeconomic conditions of the various districts varied and we distinguished between high- or very high-income areas (Sarrià-Sant Gervasi, Les Corts), medium-income areas (Ciutat vella, Eixample, Sants-Montjuic, Gràcia) and low-income areas (Horta-Guinardó, Nou Barris, Sant Andreu, Sant Martí) [20]. The rapid tests used were Determine™ HIV-1/2 for HIV, Determine™ Syphilis TP for syphilis and OraQuick™ HCV for hepatitis C. Users who tested positive were referred for confirmation, treatment and follow-up. In addition, we registered if the researcher’s profile was blocked by the app administrator. Profile blocking is usually due to complaints by users of the app.

While the programme was ongoing, we also designed a protocol for app-based interventions (Appendix 1). The face-to-face intervention included a pre-test counselling session covering sexual risk situations that evaluated risk and could lead to the recommendation of post-exposure prophylaxis (PEP), direct viral load or repeat testing after window period, based on international recommendations [21–23]. In addition, STI vaccination was carried out according to local guidelines (hepatitis A and B, and human papilloma virus).

### Intervention in Retail Pharmacies

A programme using a similar method was performed in retail pharmacies in different locations of the city, where a profile was created in Grindr® and messages were sent to nearby users. To do this, pharmacy staff were trained and were given an abbreviated protocol (Appendix 2). The following data were recorded: number of days the programme was active, the number of messages sent, number of responses obtained, the number of people visiting the pharmacy to request a test, and the number of times the pharmacy profile was blocked by the app administrator. If the profile was blocked three times, the intervention was discontinued.

### Outcome Definitions

The response rate was defined as the proportion of users responding to the message sent, among all the users who were sent a message. Acceptability was defined as the proportion of users responding favourably, among users responding to the message. Favourable responses were defined as those considered “positive”, or which generated some type of engagement, versus rejection, indifferent response, or non-response. Examples of positive responses are (translated to English): *“Thank you! All those (tests) I have made it two weeks ago, but I’ll take it into account”*, *“I am interested, how can I do it?”*. Negative responses examples are: *“Why are you telling me this? Do I look like an irresponsible person?”*. Indifferent responses were those we were unable to categorise as positive or negative, such as: *“Hello”*, *“Who are you?”*. Effectiveness was defined as the proportion of users who underwent testing among the users whom the message was sent. To identify users when they responded, the type of response, intention to get tested and attending the testing site, we created an anonymous record with a unique identifier for each contacted user.

Calculations, and subsequent analysis, were performed separately among the interventions conducted in retail pharmacies and elsewhere since the method of contact and setting differed.

### Statistical Analysis

To achieve an acceptability of 70%—as estimated by previous studies reporting this measure, [24, 25]—the required number of responses needed to obtain a 95% confidence level and a precision of 0.05 units would be 1259. The sample size was calculated using the GRANMO calculation program, version 7.12.

A descriptive univariate analysis was conducted and main variables were reported for response rate, acceptability and effectiveness. Proportions and 95% confidence intervals (CI) were calculated for categorical variables and medians and interquartile ranges (IQR) were calculated for quantitative variables. A bivariate analysis was performed for response rate, acceptability and effectiveness, using the chi-square test for categorical variables and the Mann-Whitney U test for quantitative variables. Statistical significance was set at < 0.05.

To determine the factors associated, a multivariate analysis was performed through logistic regression, and the final model was selected according to Akaike information criteria (AIC) among all possible models [26]. For the model selection, we included the variables that had a significance of > 95% in the bivariate analysis, age due to its epidemiological relevance, and district due to the study objectives. The data are expressed as adjusted odds ratios (aOR) and CI. The analysis was performed with the Stata 15® programme.

## Results

**Fig. 2:**
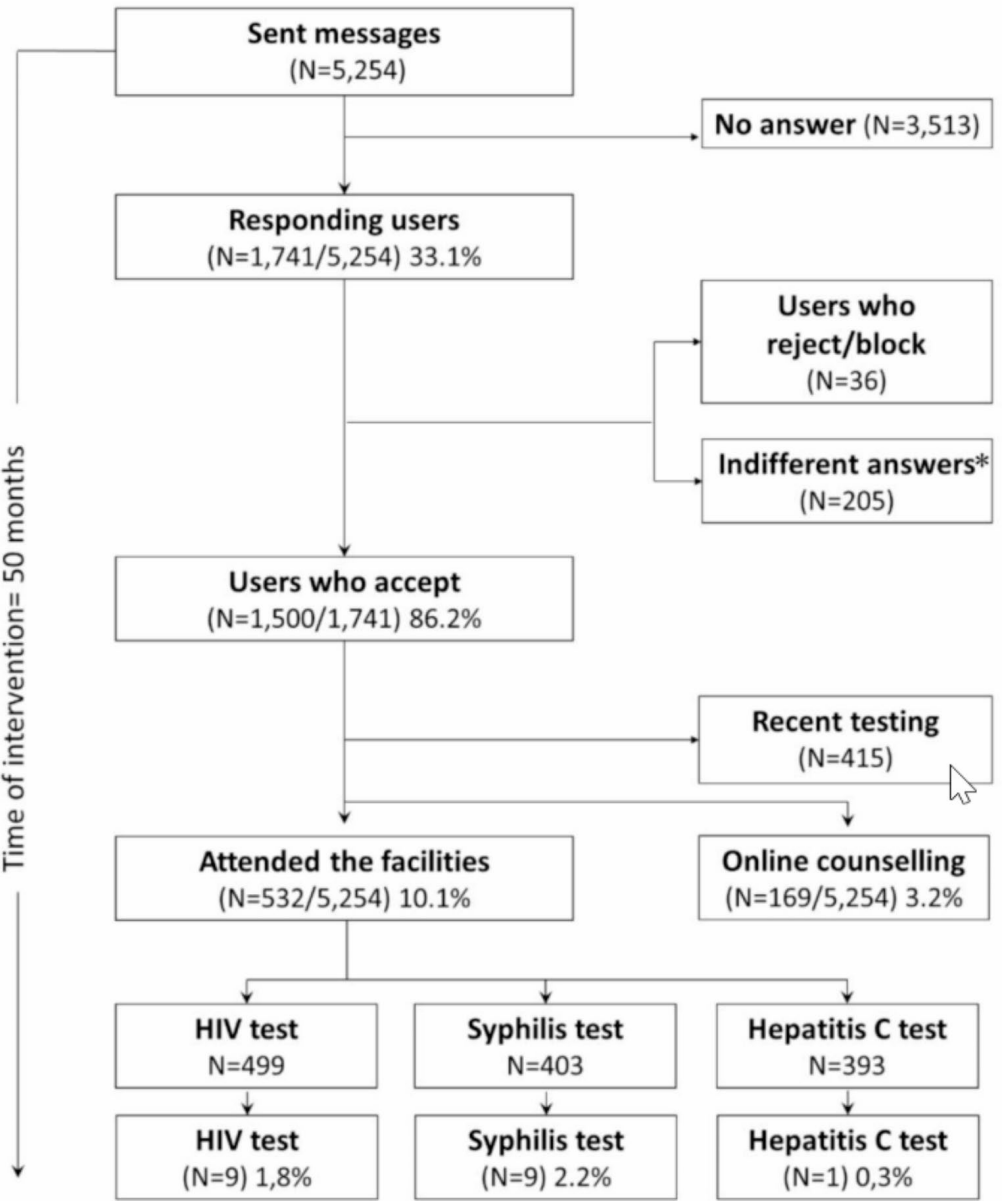
Flow chart of the main outcomes of an HIV/STI prevention programme through geolocation apps in Barcelona, Spain. *Indifferent answers were those did not qualify as positive or negative

A message was sent to 5,254 users, and responses were obtained from 33.1% (n = 1,741). Of these, 86.2% (n = 1,500) were favourable. Among these users, 415 had recently undergone testing and were consequently unsuitable for the testing intervention. A total of 532 users attended a centre to undergo a test, representing an effectiveness of 10.1% (n = 532/5,254). In the 532 tested users, 499 HIV tests, 403 syphilis tests, and 393 hepatitis C tests were performed. Among these tests, HIV infection was confirmed in 1.8% (n = 9/499), syphilis in 2.2% (n = 9/403) and hepatitis C in 0.3% (n = 1/393). In all, 3.2% (n = 169/5,254) of users responded that they would like to resolve doubts about their sexual health through the app. A flowchart of the intervention with the main outcomes is shown in Fig. 2. The researcher’s profile was blocked once in Grindr® app but was restored after contact was made with the app administrator.

The main characteristics of the intervention are shown in Table 1. Nearly half (46.2%) of the messages were sent between 14:00 and 17:00. The most widely used app was Grindr (52.2%) and the district showing the highest number of responses was Gràcia (25.9%).

Among the persons contacted, the median age given in user profiles was 32 (IQR: 26–38) years, and 91% were recently connected when the message was sent (at the same time or during the last hour).

### Response to the Intervention

In the bivariate analysis, the factors associated with a greater response to the intervention were more recent user connection (33.5% of responses; p < 0.001), the presence of a photograph in the user profile (33.4%; p < 0.001), and the districts where the intervention was performed (p < 0.001), with effectiveness being highest in Gràcia (38.5%), Nou Barris (37.6%), Sant Andreu (37.1%), and Ciutat Vella (34.9%) (Table 1). In the multivariate analysis, response was significantly associated with recent online connection to the app among users (aOR = 1.85; CI:1.39–2.46), the presence of a profile photograph (aOR = 1.34; CI:1.11–1.64), and the intervention being conducted in the districts of Ciutat Vella (aOR = 2.12; CI: 1.64–2.74), Sant Gervasi (aOR = 1.68; CI: 1.10–2.59), Gràcia (aOR = 2.50; CI: 1.93–3.24), Horta-Guinardó (aOR = 1.88; CI: 1.39–2.54), Nou Barris (aOR = 1.74; CI: 1.10–2.73), and Sant Andreu (aOR = 2.22; CI:1.40–3.51).

**Table 1:** Univariate analysis of the main variables of the prevention programme through geolocation apps in Barcelona, Spain

Variable	N	%
**Total sent messages**	5,254	
**Time of the intervention**		
< 14:00	1,374	26.1%
14:00 to 17:00	2,427	46.2%
17:00 or later	1,453	27.7%
**Application used**		
Planet Romeo	765	14.6%
Grindr	2,745	52.2%
Wapo	1,666	31.7%
Scruff	51	1.0%
Missing	27	0.5%
**District of the intervention**		
Ciudat vella	1,591	30.3%
Eixample	510	9.7%
Sants-Montjuic	580	11.0%
Sant Gervasi	172	3.3%
Gracia	1,361	25.9%
Horta-Guinardó	559	10.6%
Nou Barris	202	3.9%
Sant Andreu	140	2.7%
Sant Martí	134	2.6%
**Age** (Median [IQR])	32	[26–38]
**Time of connection**		
Connected in the last hour	4,782	91.0%
More than 1 h ago	332	6.3%
Missing	140	2.7%
**Profile photograph**		
With photo	785	14.9%
Without photo	4,438	84.5%
Missing	31	0.6%

**Table 2:** Factors associated with the level of response, acceptability, and effectiveness of the prevention programme through geolocation apps in Barcelona, Spain. Bivariate and multivariate analyses

	Response				Acceptability				Effectiveness			
Variables	Yes	No	p-value^a^	aOR^b^	Yes	No	p-value^a^	aOR^b^	Yes	No	p-value^a^	aOR^b^
**Age** (median[IQR])	32 [27–39]	32 [26–39]	0.312	1.00 [0.99–1.01]	32 [27–39]	31 [26–40]	0.373	1.01 [0.99–1.03]	30 [25–35]	32 [26–39]	0.003	0.98 [0.97–0.99]
**Time of intervention**												
< 14:00	31.40%	68.60%	0.293		87.70%	12.20%	0.433		12.30%	87.70%	< 0.001	2.47 [1.77–3.46]
14:00 to 17:00	33.60%	66.40%			86.20%	13.80%			6.10%	93.90%		1
17:00 or later	33.90%	66.10%			84.80%	15.20%			9.50%	90.50%		1.12 [0.80–1.55]
**Time of connection**												
Connected in the last hour	33.50%	66.50%	< 0.001	1.87 [1.41–2.48]	86.20%	13.80%	0.030	1.98 [1.09–3.58]	6.90%	93.10%	0.002	4.89 [1.98–12.08]
More than 1 h ago	24.10%	75.90%		1	77.50%	22.50%			2.50%	97.50%		
**Profile photograph**												
With photo	34.40%	65.60%	< 0.001	1.34 [1.11–1.64]	86.30%	13.70%	0.470		7.10%	92.90%	0.247	
Without photo	26.20%	73.80%		1	84.50%	15.50%			8.30%	91.70%		
**District of the intervention**												
Ciutat Vella	34.90%	65.10%	< 0.001	2.12 [1.64–2.74]	85.60%	14.40%	0.071	0.45 [0.19–1.08]	3.40%	96.60%	< 0.001	0.76 [0.46–1.37]
Eixample	24.90%	75.10%		1.23 [0.89–1.70]	89.80%	10.20%		0.65 [0.22–1.91]	4.20%	95.80%		0.93 [0.47–2.01]
Sants-Montjuic	23.10%	76.90%		1	92.50%	7.50%		1	5.60%	94.40%		1
Sant Gervasi	29.70%	70.40%		1.68 [1.10–2.59]	92.20%	7.80%		0.73 [0.22–1.91]	8.40%	91.60%		1.56 [0.64–4.33]
Gràcia	38.50%	61.50%		2.50 [1.93–3.24]	83.00%	17.00%		0.38 [0.16–0.91]	18.00%	82.00%		3.18 [2.07–5.42]
Horta-Guinardó	33.10%	66.90%		1.88 [1.39–2.54]	84.90%	15.10%		0.43 [0.16–1.13]	4.90%	95.10%		1.36 [0.73–2.83]
Nou Barris	37.60%	62.40%		1.74 [1.10–2.73]	88.20%	11.80%		0.39 [0.12–1.35]	6.60%	93.40%		1.83 [0.79–4.81]
Sant Andreu	37.10%	62.90%		2.22 [1.40–3.51]	92.30%	7.70%		0.91 [0.21–3.95]	14.60%	85.40%		5.08 [2.59–11.17]
Sant Martí	27.60%	72.40%		1.58 [0.97–2.56]	89.20%	10.80%		0.69 [0.16–3.05]	19.20%	80.80%		5.83 [3.09–12.42]

a = U Mann-Whitney test for numerical variables and Chi-square test for categorical variables

b = Adjusted Odd Ratios

IQR = Interquartile Range

### Acceptability of the Intervention

After bivariate analysis, the only factor associated with greater acceptability was the time of the user’s most recent online connection (86.2% of acceptability; p = 0.030). No significant differences were found among the remaining factors (Table 1). After multivariate adjustment, greater acceptability was significantly associated with recent user connection to the app (aOR = 1.98; CI:1.09–3.58) and performance of the intervention in the district of Gràcia (aOR = 0.38; CI:0.16–0.91).

### Effectiveness of the Intervention

After bivariate analysis, greater effectiveness was associated with younger age reported in users’ profiles (median 30 years old; p = 0.003); the time when the message was sent, before 14:00 (12.3% effectiveness) or after 17:00 (9.5%) (p < 0.001); the time of the user’s most recent connection (6.9%; p = 0.002); and the district where the intervention was performed (p < 0.001), with effectiveness being highest in Sant Martí (19.2%), Gràcia (18.0%) and Sant Andreu (14.6%) and lowest in Ciutat Vella (3.4%) (Table 1). After multivariate adjustment, greater effectiveness was significantly associated with younger age (aOR = 0.98; CI:0.97–0.99), sending of messages before 14:00 (aOR = 2.47; CI: 1.77–3.46), recent user connection to the app (aOR = 4.89; CI:1.98–12.08) and performance of the intervention in Gràcia (aOR = 3.18; CI:2.07–5.42), Sant Andreu (aOR = 5.08; CI:2.59–11.17), and Sant Martí (aOR = 5.83; CI:3.09–12.42).

### Retail Pharmacies

Of 37 pharmacies available in the city of Barcelona, the intervention was conducted in 7, and complete data was provided by 3. In the latter, the intervention lasted for a mean of 35 days, 434 messages were sent, and the response rate was 18.2% (n = 79). Finally, 12 people were tested, representing an effectiveness of 2.76% (n = 12/434). In the remaining 4 pharmacies, the profile was blocked by the app administrators and no interventions could be made. Consequently, interventions in retail pharmacies were cancelled due to the lack of feasibility.

## Discussion

This study demonstrates that the preventive intervention performed through GSN apps obtained a high response rate and good acceptability and effectiveness among users contacted over a 4-year period. Moreover, this is the first study of this kind of intervention to determine the factors associated with specific indicators, as response rate, acceptability and effectiveness, providing essential knowledge for the design of future interventions.

The high response rate shows the viability of implementing programmes using these apps as a sexual health strategy among their users in this setting. Previous studies similar to our own have reported user response rates of up to 26.5%, but in the context of study recruitment [27]. Strategies measuring response rates often occur within the context of developing new platforms, unlike this intervention, which created personal profiles in already existing apps [8, 28, 29]. In this regard, we believe that these profiles facilitate an enhanced response as they are created in spaces already known to users and do not require them to perform actions different from their daily acts or to use device storage. On the other hand, because these types of intervention are new, they may generate distrust among users who might be reluctant to reveal confidential information on their sexual health. These factors, the already known spaces and distrust of new elements, may create a disposition in users, and these match to the concepts of control and subjective norms described within the theory of planned behaviour, which describes an individual´s intention to participate in a behaviour in a specific time and place [30].

The high acceptability of unsolicited messages in a setting strongly linked to sexuality suggests the need for users to have a reference point for sexual health in these contexts [31, 32]. Although spaces for information on sexual health are provided in some apps, such as Grindr, there are no options for cybereducation, or online sexual health counselling delivered by trained peers or health care workers in commercial GSN apps. Other studies exploring receptiveness to possible interventions among app users have reported an effectiveness of up to 70% [24]. Therefore, smartphone app-based prevention has been described in this context and could be replicated in other contexts, such as other cities or rural areas, since the characteristics of users of these apps have been reported to be similar in other parts of the world [8, 33].

The effectiveness achieved in this study is a positive element and suggests the possibility of replicating the programme in other settings. We found no similar data about effectiveness in other programmes with these characteristics. We were only able to compare our data with those of some studies reporting the recruitment rate of app users to participate in studies in exchange for remuneration, such as the rate obtained in the study by Holloway et al., which was 12.8% [24]. Due to the geographical scope of these apps and lower concentration of GBMSM users, these interventions could be even more effective in rural areas, where a larger territory could be covered.

### Retail Pharmacies

The intervention carried out by retail pharmacies obtained a low response rate and effectiveness. Moreover, because of continual blocking by app administrators, this type of intervention is not feasible in this context. These results could be influenced by two factors. First, the presence of the pharmacy in the app could be seen as a threat to privacy among users embarrassed to ask for a test in person or who felt that the staff might be judgmental. Second, the pharmacy could be perceived as a for-profit organization attempting to sell a product, thus increasing income by selling tests, even though the price of testing was lower than usual in pharmacies.

### Factors Associated with Response Rate, Acceptability and Effectiveness

The effectiveness of the intervention was associated with younger user age. This finding could be related to familiarity or normalization of the use of apps for multiple tasks in the youngest populations [8, 34]. However, we believe that, with the greater incorporation of technologies in all age ranges, this variable may become less important in future.

Effectiveness was also associated with the timing of message sending and was higher with messages sent before 14:00. This could be a local factor that could differ in other contexts. Therefore, it would be advisable to adapt the timing of message sending in any future programmes when they are underway. That means it is necessary to adapt preventive programs to specific contextual characteristics, conducting simultaneous evaluation and implementation in order to improve outcomes, as has been reported previously [35].

One of the factors most strongly associated with the response rate, acceptability and effectiveness was recent use of apps by users. Given that most testing in this intervention occurred within 48 h of message sending, a possible explanation for this association could be the emphasis on immediacy within GSN apps. These apps are typically used by seeking instant encounters who are more likely to quickly transition to a different location [36].

Despite being a local factor, the districts where the intervention was conducted generate valuable information on the geospatial characteristics of an app-based intervention, as well as on the particular contexts of the territorial diversity of the city. In Barcelona, economic indicators have been created by districts, revealing significant inequalities [20]. Thus, it can be deduced that the districts with the highest response rate and greatest effectiveness, apart from the district of Gràcia, have a lower socioeconomic level and fewer available services offering testing and prevention targeting vulnerable populations [37]. This indicates that this type of intervention is even more useful in disadvantaged areas and those with less access to sexual health services, as may be the case in rural areas where this type of intervention has been reported to be acceptable to GBMSM [33]. The district of Gràcia, which has a higher socioeconomic level and greater availability of STI screening services, had a higher response rate and greater effectiveness. This finding may be due to its over-representation in the given that most of the messages were sent from our centre in this district. In addition, among the centers included in this study, the center in Gracia was that which remained operational for the longest time within the study period, making it easily recognizable to nearby users.

Moreover, this intervention was able to provide preventive services and sexual health counselling to a substantial number of persons through the in-app chat feature. This activity, previously described as “reaching out online”, has been shown to be effective as an alternative to peer counselling in leisure venues and centres dedicated to sexual health counselling [38].

A substantial number of attended persons tested positive for STI, indicating the opportuneness of this type of intervention in places concentrating the population at risk for STI. In 2010, the ECDC defined interventions as highly effective if more than 1% of persons tested had a positive result [39].

### Limitations and Strengths

One of the limitations of this study is its cross-sectional design, which cannot demonstrate causality, only associations. GSN app-based recruitment could fail to reach the GBMSM population not using these platforms, although these individuals were not included in the aim of this study. Moreover, offering rapid HIV and syphilis testing might not appeal to persons already diagnosed with HIV, users of HIV pre-exposure prophylaxis, and those with prior syphilis infection, which should be taken into account when evaluating future interventions. The response rates, acceptability and effectiveness reported here refer to a specific area the city of Barcelona, where there is strong awareness of LGBT + sensitivities and needs, and consequently levels could be lower in areas with less receptiveness or greater homophobia. Another limitation is the timing of the intervention (between 8:00 and 19:00 h), as some app users connect at other times and may have different characteristics.

GSN app-based interventions may have technical limitations, such as the radius of visibility or the number of users who can be reached, as well as the limitations related to acceptability among users. Their use poses ethical dilemmas related to the mechanisms for obtaining data by users due to the absence of consent in app-based interventions. One of the limitations specific to using a virtual setting is the inability to guarantee the confidentiality of the data obtained, which is owned by a private company [40]. Likewise, user recruitment should encompass individual variability within the GBMSM collective, which could be reflected in the various platforms. This variety of apps is related to the distinct goals among the users of each app and their different sociodemographic characteristics, generating differences in the response rate, acceptance and effectiveness of interventions [41].

Among the strengths of the study is the standardised use of indicators, as response rate, acceptability and effectiveness, over a prolonged period, allowing greater representativeness of the local reality. The geographical representativeness of the city and the large number of users contacted enhanced the ability of this study to analyse the topic investigated. Moreover, the app-based intervention allowed counselling and education strategies to be performed in real time, making it a highly opportune tool for prevention and screening.

The performance of this intervention indirectly prompted the creation of a space dedicated to the sexual health of key collectives directed by the epidemiological surveillance service of the city. This opportunity allowed links to be forged with other community and healthcare services, transforming the intervention into one of the sexual health services in the area. We believe that these interventions and secondary integration processes strengthen the response of these services to future sexual health needs.

### Recommendations for App-Based Interventions

Factors that could be related to the success of the intervention are the geographical proximity of researchers and users, the possibility of testing shortly after the first contact and its rapid results, the absence of a charge for the service, its performance as a community service rather than as a clinical procedure, the provision of sexual counselling, confidentiality, the direct approach to users, and the personal and friendly attitude of the intervenor. These factors have been identified as the preferred attributes in STI testing services among GBMSM [42]. Moreover, elements can be drawn from m-Health studies, such as apps designed for prevention and sexual health promotion. These have been effective with elements such as adaptation to user behaviours or the gamification of the design [43–45].

Some app-based interventions have raised ethical questions, mainly concerning confidentiality and the use of data and metadata for research. These considerations should be borne in mind before an activity of this type is conducted, whether for research or intervention purposes [40].
